# Technology and democracy: a paradox wrapped in a contradiction inside an irony

**DOI:** 10.1017/mem.2021.7

**Published:** 2021-12-09

**Authors:** Stephan Lewandowsky, Peter Pomerantsev

**Affiliations:** 1 School of Psychological Science, University of Bristol, Bristol BS8 1TU, UK; 2 SNF Agora Institute, Johns Hopkins University, Baltimore, Maryland

**Keywords:** Democracy, populism, misinformation, social media, Internet

## Abstract

Democracy is in retreat around the globe. Many commentators have blamed the Internet for this development, whereas others have celebrated the Internet as a tool for liberation, with each opinion being buttressed by supporting evidence. We try to resolve this paradox by reviewing some of the pressure points that arise between human cognition and the online information architecture, and their fallout for the well-being of democracy. We focus on the role of the attention economy, which has monetised dwell time on platforms, and the role of algorithms that satisfy users’ presumed preferences. We further note the inherent asymmetry in power between platforms and users that arises from these pressure points, and we conclude by sketching out the principles of a new Internet with democratic credentials.

The mission of *Memory, Mind & Media* is to document and explore the impact of media and technology on human, social and cultural remembering and forgetting. In this article, we set out the key challenges for the field, and hence the core issues and ideas for the journal, through the lens of the unique cognitive pressure points that create tension between the online information ecology and democratic discourse and governance.

Numerous indicators suggest that democracy is in retreat globally (Freedom House 2020; Lührmann and Lindberg 2020). Even countries that had been considered stable democracies, such as the United States (US) and the United Kingdom (UK), have recently witnessed events that are incompatible with democratic governance and the rule of law, such as the armed assault on the U.S. Capitol in 2021 and the unlawful suspension of the British parliament in 2019.

Although the symptoms and causes of democratic backsliding are complex and difficult to disentangle, the Internet and social media are frequently blamed in this context. For example, social media has been identified as a tool of autocrats (Deibert 2019).

Empirical support for this assertion arises from the finding that the more committed autocratic regimes are to prevent an independent public sphere, the more likely they are to introduce the Internet (Rød and Weidmann 2015). In Western democracies, recent evidence suggests that social media can cause some anti-democratic political behaviours ranging from ethnic hate crimes to voting for populist parties (Bursztyn et al 2019; Müller and Schwarz 2019; Allcott et al 2020; Schaub and Morisi 2020). Social media have also been blamed for increasing political polarisation (Van Bavel et al 2021). Some scholars have openly questioned whether democracy can survive the Internet (Persily 2017).

In the opposing corner, social media has been heralded as ‘liberation technology’ (Tucker et al 2017), owing to its role in the ‘Arab Spring’, the Iranian Green Wave Movement of 2009, and other instances in which it mobilised the public against autocratic regimes. Similarly, protest movements in the US, Spain, Turkey, and Ukraine rely on social media platforms for the coordination of collective action and to transmit emotional and motivational messages (Jost et al 2018). A recent field experiment in an ethnically highly polarised society, Bosnia and Herzegovina, found that people who continued to use Facebook reported greater outgroup regard than a group that voluntarily deactivated Facebook for the same time period (Asimovic et al 2021).

## The fundamental paradox

This is the fundamental paradox of the Internet and social media: They erode democracy and they expand democracy. They are the tools of autocrats and they are the tools of activists. They make people obey and they make them protest. They provide a voice to the marginalised and they give reach to fanatics and extremists. And all of these conflicting views are seemingly supported by analysis or empirical evidence, rendering resolution of this paradox difficult.

We have proposed elsewhere that to understand this basic paradox, we must examine the unique pressure points that arise when human cognition is let loose on the Internet (Kozyreva et al 2020; Lewandowsky et al 2020; Lorenz-Spreen et al 2020). The interaction between fundamental human cognitive attributes and the architecture of the information ecology have created a perfect storm for democracy. Here, we focus on a subset of these pressure points and highlight how they, in turn, also contain intrinsic ironies and paradoxes.

## The attention economy

Our attention has been commodified (Wu 2017). When we use a ‘free’ product online, we are the product. The more time we spend watching YouTube videos or checking our Facebook newsfeed, the more advertising revenue is generated for the platforms. This commodification of attention is an inescapable driver of online behaviour that has several contradictory consequences. On the positive side, the fact that dwell time online has become revenuegenerating currency has enabled the creation of a vast array of – seemingly – free services. YouTube *is* free to use and provides nearly unlimited entertainment options. Google offers a suite of tools beyond its search engine, from email to document creation, that support countless endeavours free of charge. Facebook permits us to stay in touch with friends and family, and we can use WhatsApp to make video calls with people all around the world at no cost. The array of free services available online is impressive by any measure.

But those free services are not truly free – on the contrary, they incur considerable costs that are often external to the interactions we intentionally engage in. One implication of the conversion of dwell time into revenue-generating currency is that the platforms will naturally try to present us with captivating information to retain our attention. This commercial incentive structure is potentially problematic because people are known to attend to news that is predominantly negative (Soroka et al 2019) or awe-inspiring (Berger and Milkman 2012). People also preferentially share messages couched in moral-emotional language (Brady et al 2017). It is unsurprising, therefore, that ‘fake news’ and misinformation have become so prevalent online because false content – which by definition is freed from factual constraints – can exploit this attentional bias: misinformation on Facebook during the 2016 U.S. presidential campaign was particularly likely to provoke voter outrage (Bakir and McStay 2018) and fake news titles have been found to be substantially more negative in tone, and display more negative emotions such as disgust and anger, than real news titles (Paschen 2019). The flood of disinformation and online outrage is, therefore, arguably a price we pay for the ‘free’ services provided by the platforms.

Although human attentional biases did not suddenly change just because the Internet was invented – the adage that ‘when it bleeds, it leads’ is probably as old as journalism itself – web technology has turbo-charged those biases in at least two ways. First, the sheer quantity of information online has measurable adverse consequences for our ‘collective mind’ and societal memories. Whereas in 2013 the most popular hashtags on Twitter remained popular for 17.5 h, by 2016 a hashtag’s life in the limelight had dropped to 11.9 h (Lorenz-Spreen et al 2019). A similar decline in our collective attention span was observed for Google queries and movie ticket sales (Lorenz-Spreen et al 2019). It is unsurprising that political accountability will become more difficult in societies with a shorter attention span: if a leader’s original transgression is forgotten in a few hours, the public appetite for accountability is unlikely to be lasting (Giroux and Bhattacharya 2016). Even highly consequential events can seemingly disappear without leaving much of a trace: When British Prime Minister Boris Johnson prorogued (ie, shut down) Parliament on 24 August 2019 to escape further scrutiny of his Brexit plans, public interest was initially intense. After this prorogation was found to be unlawful by the Supreme Court on 24 September 2019, public interest in the issue, as measured by Google Trends, dissipated by 93% within 5 days. Within 2 months, public interest in prorogation returned to the near-zero level observed before the prorogation, when hardly anyone in Britain even knew the term ‘prorogation’ existed.^1^ Johnson went on to win an election a few months later by a landslide.

The problems arising from a shortened attention span are compounded by the fact that information overload generally makes it harder for people to make good decisions about what to look at, what to spend time on, what to believe, and what to share (Hills et al 2013; Hills 2019). Choosing a paper to purchase at a newsstand requires a single decision. Our Twitter feed or Facebook newsfeed confronts us with the need for a multitude of microdecisions for every article or post. Although these repeated micro-decisions open the door to greater diversity in our news diet, they also increase the probability that at least some of our chosen sources fail to be trustworthy. Worse yet, information overload can also contribute to polarisation and dysfunctional disagreement between well-meaning and rational actors (Pothos et al 2021). That is, despite their good-faith efforts, overload may prevent actors from forming compatible mental representations of complex problems. Excessive complexity mandates a simplification of representations, and this, in turn, necessarily introduces potential incompatibilities between actors that may result in irresolvable disagreement (Pothos et al 2021).

The second turbocharger of human cognitive biases by online technologies relies on the exact measurement of our responses to information. Facebook has access to our every click while we are on the platform, and Facebook can use that information for continual personalised refinement of our information diet through the platform’s algorithms.

## The Jekyll and Hyde of the algorithm

Most of the information we consume online is shaped and curated by algorithms. YouTube, by default, keeps playing videos we are presumed to like based on inferences by its recommender system. Facebook’s newsfeed is curated by a sophisticated algorithm, and Google’s search results are customised according to numerous parameters. Algorithms are an essential tool to harness the abundance of information on the web: Googling ‘Georgia’ should return different results in Atlanta than in Tbilisi, and without such intelligent filtering, useful information would most likely remain inaccessible. Algorithms can also help us satisfy our preferences, for example, when recommender systems help us find movies, books, or restaurants that we are likely to enjoy (Ricci et al 2015). It is unsurprising, therefore, that the public is mainly appreciative of algorithms and customisation in those contexts (Kozyreva et al 2021).

There are, however, several darker sides to algorithms. The first problem is that algorithms ultimately serve the interests of the platforms rather than the users. An ironic consequence of this is that in the relentless pursuit of increasing dwell time, algorithms may eagerly satisfy our presumed momentary preferences even if that reduces our long-term well-being. In the same way that strategically placed junk food in the supermarket can satisfy our cravings while also propelling an obesity epidemic, algorithms may satisfy our momentary desire for emotional engagement while contributing to the formation of sealed anti-democratic communities (Kaiser and Rauchfleisch 2020). Unconstrained preference satisfaction may ironically create fractionated and polarised societies (Pariser 2011).

The second problem with algorithms is that their design and operation are proprietary and not readily subject to public scrutiny. Most algorithms operate as ‘black boxes’ where neither individual users nor society, in general, know why search results or social media feeds are curated in a particular way (Pasquale 2015). At present, knowledge about the algorithms can only be obtained by ‘reverse engineering’ (Diakopoulos 2015), that is, by seeking to infer an algorithm’s design based upon its observable behaviour.

Reverse engineering can range from the relatively simple (eg, examining which words are excluded from auto-correct on the iPhone; Keller 2013) to the highly complex (eg, an analysis of how political ads are delivered on Facebook; Ali et al 2019). Reverse engineering has uncovered several problematic aspects of algorithms, such as discriminatory advertising practices and stereotypical representations of Black Americans in Google Search (Sweeney 2013; Noble 2018) and in the autocomplete suggestions that Google provides when entering search terms (Baker and Potts 2013). At the time of this writing, a Facebook whistle-blower revealed further information about how content is being highlighted on the platform. It transpired that any content that made people angry – which was disproportionately likely to include misinformation, toxicity, and low-quality news – was given particular prominence in people’s newsfeed. Facebook thus ‘systematically amped up some of the worst of its platform, making it more prominent in users’ feeds and spreading it to a much wider audience’ (Merrill and Oremus 2021).

The opacity of algorithms allows platforms to drench users in information that may be detrimental to democratic health. Even ignoring the specifics of content, algorithmic opacity also contributes to a general imbalance of power between platforms and users that can only be unhealthy in a democracy.

## The asymmetry of power

The platforms know much about their users – and even about people who are not on their platforms (Garcia 2017) – and deploy that knowledge to shape our information diets. By contrast, citizens know little about what data the platforms hold and how these data are used (Lorenz-Spreen et al 2020). For example, Facebook ‘likes’ can be used to infer our personality through machine learning with considerable accuracy (Youyou et al 2015). Knowledge of just a few likes raises machine-learning performance above that of work colleagues, and with knowledge of 300 likes, the performance of the machine exceeds that of one’s spouse (Youyou et al 2015). In stark contrast to the power of machine learning, a substantial share of people does not even know that their Facebook newsfeed is curated based on personal data (Eslami et al 2015; Rader and Gray 2015; Powers 2017), with estimates of this lack of awareness ranging from 27 to 62.5 per cent.

Asymmetry in knowledge translates into an asymmetry of power: To keep others under surveillance while avoiding equal scrutiny oneself is the most important form of authoritarian political power (Balkin 2008; Zuboff 2019). Similarly, to know others while revealing little about oneself is the most important form of commercial power in an attention economy. When Facebook recently shut down the accounts of researchers who were studying how misinformation spreads and how users are targeted on the platform (Edelson and McCoy 2021), it did not do so to preserve users’ privacy as it claimed. That claim was quickly and thoroughly rejected by the Federal Trade Commission. Facebook shut down the researchers to preserve its asymmetrical power advantage by preventing an examination of how it operates. It is this power asymmetry that renders the freedom and choice offered by the Internet largely illusory.

## The illusion of freedom and choice

Everyone gets a voice on the Internet. On the positive side of the ledger, there is evidence that access to the Internet leads to enhanced transparency and reduction of corruption. In a cross-national analysis of 157 countries, Starke et al (2016) showed that Internet access was associated with a significant reduction in official corruption. On the more negative side of the ledger, a single tweet can trigger a cascade of adverse events. The ‘pizzagate’ affair of 2016 was triggered by a baseless accusation that the Democratic party was operating a paedophilia ring out of the basement of a pizza parlour in Washington, D.C. This conspiracy theory was eventually picked up by mainstream media, and ultimately an armed individual entered the pizza parlour and fired shots inside in search of a (nonexistent) basement (Fisher et al 2016).

The ambivalent consequences of unfettered access to the Internet are amplified by the opportunities offered for manipulation through targeted advertising. All advertising and political speech seek to persuade. Manipulation differs from persuasion by furtively exploiting a target’s weaknesses and vulnerabilities to steer their behaviour in a desired direction (Susser et al 2019). The fact that Facebook ‘likes’ permit inferences about a user’s personality (Youyou et al 2015), combined with the fact that advertisers can select target audiences based on those likes (coded as users’ interests), offers an opportunity for targeted manipulation on a global scale and without any transparency. Research suggests that single individuals or households can be targeted with messages using Facebook’s ad delivery services (Faizullabhoy and Korolova 2018). Although the effectiveness of such ‘microtargeting’ of messages is subject to debate (eg, Matz et al 2017 vs. Eckles et al 2018), there is no question that targeting of political messages at individuals (or small numbers of individuals) facilitates the dissemination of disinformation because political opponents cannot know what is being said and hence cannot rebut false information (Heawood 2018). Similarly, microtargeting allows politicians to make multiple incompatible promises to different audiences without anyone being able to track and point out those incompatibilities (Heawood 2018). A recent pertinent example arose during the German parliamentary election in September 2021. The Liberal Democratic Party (FDP) was found to target Facebook users with ‘green’ interests with a message that identified the party with ‘more climate protection’ through a regulatory upper limit on CO_2_ emissions. At the same time, the FDP targeted frequent travellers on Facebook with an ad that promised ‘no state intervention or restrictions of freedom or prohibitions’ to address climate change.^2^

Unsurprisingly, the public overwhelmingly rejects this type of manipulative targeting (Kozyreva et al 2021).^3^

Everyone may get a voice on the Internet. But everyone is also exposed to a cacophony of voices whose origin may be obscured and that may seek to manipulate rather than inform. The power to design and deliver manipulative messages that form our society’s collective memory rests with advertisers and platforms rather than citizens. For now at least, the freedom and choice offered by the Internet, therefore, remains largely illusory.

## Building a better Internet

Our preceding analysis illustrates the fundamental paradox of the online media environment: On the one hand, there is more information than ever before, but we know less than ever about how that information is produced, targeted, organised, and distributed. Citizens do not know why algorithms show them one thing and not another, or which of their own data are being used to target them and why. Citizens have little way of knowing about the vast social engineering experiments tech companies play with as they fiddle with their algorithms. Citizens do not even know if their basic rights are being infringed by manipulative algorithms and advertisers. We believe that democratic societies would never have consented to any of those consequences of the Internet if they had been known ahead of time or if the Internet had been designed with those attributes in mind. It is only because the Internet evolved, one technological innovation and one tweak to an algorithm at a time, that democracies are only now realising what they are confronting.

What, then, should the online experience be like for a person in a democracy? How can be design and build a better Internet? We have both been involved in developing specific recommendations for a better Internet (eg, Kozyreva et al 2020; Lewandowsky et al 2020; Lorenz-Spreen et al 2020; Applebaum and Pomerantsev 2021). Here, we focus on one aspect only, namely the power asymmetry between platforms and users and how it might be redressed.

In an Internet with democratic credentials, users would be able to understand which of their own data have been used to target them and why. Users would know why algorithms show them one thing and not another. During elections, people would immediately understand how different campaigns target different people with different messages, who is behind campaigns, and how much they spend.

Online anonymity is a basic right. People should be allowed to ‘wear a mask’ online for reasons of safety and many others. But the receiver of information should also have the right to understand whether they are being targeted by a real person (whether anonymous or not), or by a political campaign, a corporation, or a state that is pretending to be a real person. ‘Troll farms’, bot nets, and other forms of mass coordinated inauthentic activity should be clearly identified as such.

An empowered online citizen would also have far greater control over their own data and would be able to regulate how others use it. There may be instances where, for example, one might be comfortable with sharing one’s data with a national health service.

But there should be strict guardrails that do not allow user data to be used further by data brokers.

And as individuals should have more oversight and control over the information environment all around them, so should the public have greater oversight and control over tech companies in general. The public need to be able to understand what social engineering experiments the companies tinker with, what their impacts are, and how the tech companies track the consequences of these experiments.

Likewise, algorithmic transparency is essential. This does not mean that companies have to reveal their proprietary source code. They do, however, need to explain the purpose of adjustments they make to their algorithms, and the changes these bring about. If algorithms infringe on people’s rights, such as in cases where algorithms produce advertising that disadvantages minorities, the public need to have oversight over what the companies are doing to rectify these discriminatory practices. Such algorithmic transparency needs to be backed up with regulatory teeth: regulators should have the right to spot-check how companies are continually analysing and mitigating negative effects of their own design decisions.

But regulation needs to go beyond just mitigating the bad and setting standards. It needs to encourage ‘the good’ too. We must design regulations that encourage the development of ‘civic tech’; that is, technology that is meant to benefit individuals and strengthen democratic processes. Such technology would be created in the public interest, and not driven by short-term profit motives to extract people’s personal data and then sell it on.

As Ethan Zuckerman of the University of Massachusetts argues^4^, we are in a similar place in the development of the Internet as we were with radio at the start of the 20th century. Back in the 1920s, in the UK, Lord Reith fought for the existence of public interest broadcasting to balance the polarising impact of press barons and the rising power of radioenhanced dictatorships. The result was the creation of the BBC. What would be the online equivalent of that today? We do not know. This illustrates the magnitude of the task ahead. It may be daunting, but that should concern us less than the conflict between current technologies and democracy that is driven, in part, by known limitations of human attention, memory, and cognition. The mission of *Memory, Mind & Media* is precisely aimed at those limitations and conflicts, and the journal is, therefore, poised to make a contribution to what we consider the defining political battle of the 21st century – the battle between technological hegemony and survival of democracy.

## Notes

1 https://trends.google.com/trends/explore/TIMESERIES/1635669000?hl=en-GB\&tz=0\&date=today+5-y\&geo=GB\&q=prorogation\&sni=3.

2 https://targetleaks.de/.

3 Recent transparency measures (eg, Facebook’s ‘ad library’) are insufficient to analyze parties’ expenditure on microtargeting and what content has been shown (Dommett and Power 2019). The ad library is also missing more than 100,000 political ads (Edelson and McCoy 2021). This difficulty is likely to persist because ads on Facebook are delivered by a continually evolving algorithm, known as AdTech, that auctions off ads on a second-to-second basis based on live analysis of user data (Ali et al 2019).

4 https://www.umass.edu/spp/news/case-digital-public-infrastructure.
